# Exosomal-miR-1184 derived from mesenchymal stem cells alleviates cisplatin-associated acute kidney injury

**DOI:** 10.3892/mmr.2022.12757

**Published:** 2022-05-31

**Authors:** Jinshi Zhang, Wenfang He, Danna Zheng, Qiang He, Mingming Tan, Juan Jin

Mol Med Rep 24: 795, 2021; DOI: 10.3892/mmr.2021.12435

Subsequently to the publication of this paper, the authors have noticed that Fig. 5 was published with inadvertent errors; essentially, the y-axis label in Fig. 5D should have read “TNF-α” instead of “IL-1β” and conversely, in Fig. 5E, the y-axis label should have read “IL-1β” instead of “TNF-α”.

The revised version of Fig. 5, with Figs. 5D and E now labelled correctly, is shown below. Note that these errors did not significantly affect either the results or the conclusions reported in this paper, and all the authors agree to this corrigendum. Furthermore, the authors thank the Editor of *Molecular Medicine Reports* for allowing them the opportunity to publish this corrigendum, and apologize to the readership for any inconvenience caused.

## Figures and Tables

**Figure 5.**
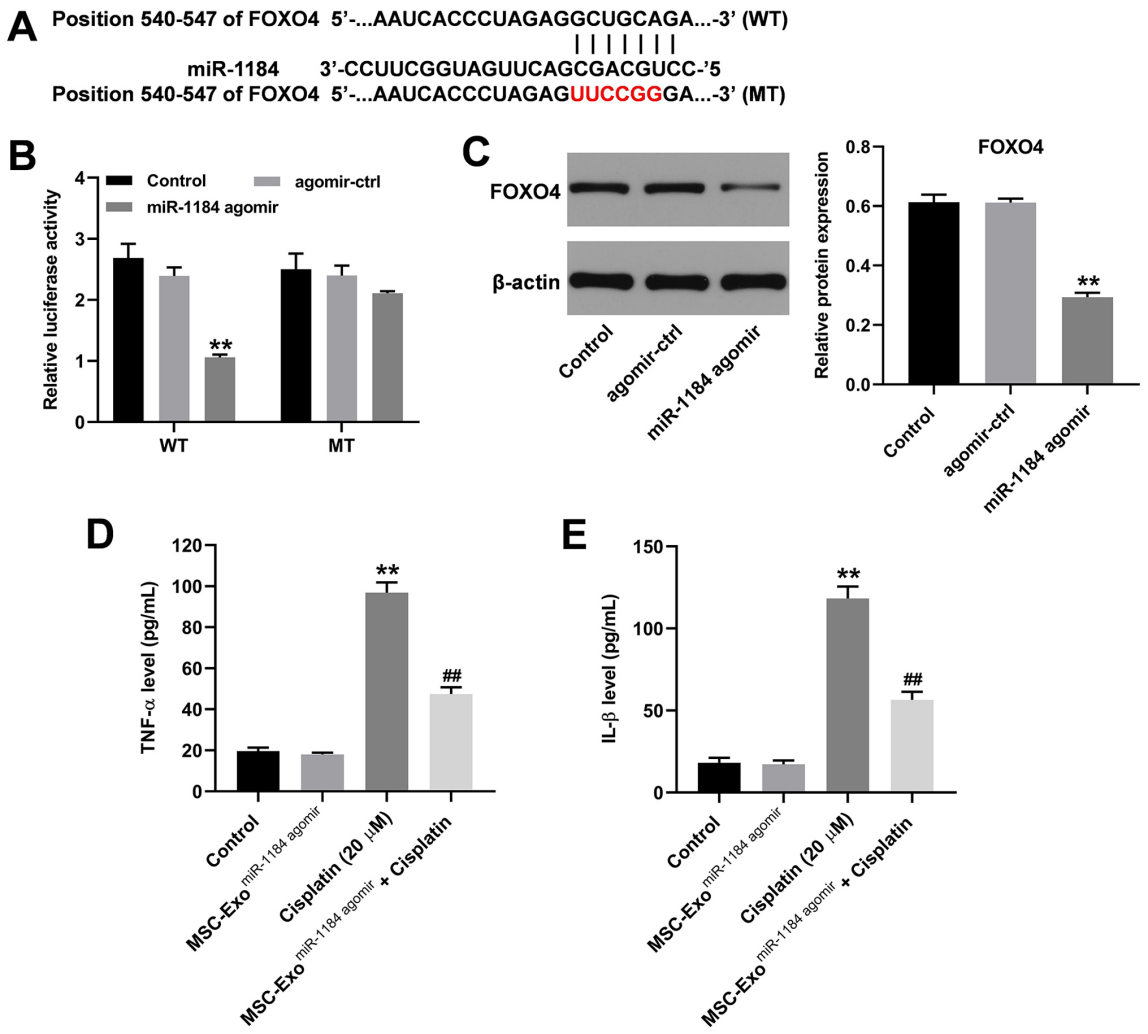
miR-1184 directly targets FOXO4 in HK-2 cells. (A) The target of miR-1184 was predicted by TargetScan and miRDB. (B) The relative luciferase activity was detected by dual-luciferase reporter assay. (C) HK-2 cells were transfected with NC or miR-1184 agomir. The expression of FOXO4 in HK-2 cells was detected by western blotting. The relative expression was semi-quantified by normalization to β-actin. (D) The level of IL-1β in supernatants of HK-2 cells was tested by ELISA. (E) The level of TNF-α in supernatants of HK-2 cells was tested by ELISA. **P<0.01 vs. control; ^##^P<0.01 vs. cisplatin. miR, microRNA; FOXO4, forkhead box O4; NC, negative control; WT, wild-type; MT, mutant; MSCs, mesenchymal stem cells; MSC-ExomiR-1184 agomir, exosomes derived from miR-1184 agomir-treated MSCs.

